# Safety Recommendations and Medical Liability in Ocular Surgery during the COVID-19 Pandemic: An Unsolved Dilemma

**DOI:** 10.3390/jcm9051403

**Published:** 2020-05-09

**Authors:** Pietro Emanuele Napoli, Matteo Nioi, Ernesto d’Aloja, Maurizio Fossarello

**Affiliations:** 1 Department of Surgical Science, University of Cagliari, Eye Clinic, via Ospedale 46, 09124 Cagliari, Italy; maurizio.fossarello@gmail.com; 2 Department of Clinical Sciences and Public Health, University of Cagliari, Forensic Medicine Unit, 09124 Cagliari, Italy; nioimatteo@gmail.com (M.N.); ernestodaloja@gmail.com (E.d.A.); 3 Clinica Oculistica, San Giovanni di Dio Hospital, Azienda Ospedaliera Universitaria di Cagliari, 09124 Cagliari, Italy

**Keywords:** COVID-19, ocular surgery, eye surgery, coronavirus, SARS-CoV-2, recommendations, medical liability, eye, guidelines

## Abstract

Ocular surgery is one of the most performed medical procedures in the world. Its limitation or suspension, recursively extended over time, could be associated with a significant increase in the number of blind people worldwide. Indeed, cataract causes more than half of all cases of visual impairment in those countries with limited availability of means for performing eye operations (e.g., Africa or India). In this scenario, the pandemic of coronavirus disease 2019 (COVID-19) quickly resulted in the suspension or sharp reduction of various ophthalmic activities considered non-urgent, including lens replacement surgery or some intraocular injections. Despite the imperative need to continuously practice eye operations to avoid the abovementioned problems, there are currently little-shared and vague recommendations among the various countries on safety in operating rooms (for health care workers and patients) and poor legal protection for surgeons (potentially transmitting the COVID-19 infectious agent). Herein, we individuated and discussed some critical points in safety recommendations and medical liability. A paradigm shift for ocular surgery during the COVID-19 era is now mandatory. While telemedicine has been able to solve some problems in clinical ophthalmology, the lack of adequate health and legal protection for surgeons and patients may result in an excessive reduction in the volume of surgical interventions during a pandemic era and the immediately following period, thus determining inability to ensure health care to all patients.

## 1. Introduction

Eye surgery is among the most performed therapeutic procedures in the world for its efficacy, safety, and demand (e.g., cataract removal) [1]. With the inactivity of operating theaters, eye diseases would lead to legal or complete blindness in a significant number of people around the globe (e.g., for neovascular macular degeneration or retinal detachment).

Specifically, cataract causes more than half of all cases of visual impairment in those countries with limited availability of means for performing eye surgery (e.g., Africa or India). In fact, cataract surgery is one of the most common medical interventions in Western countries where all the necessary devices are available. The prolonged suspension of this effective treatment would determine a drastic increase in the number of cases of blindness both in the Western world and in the least developed or developing countries.

In this scenario, the pandemic of coronavirus disease 2019 (COVID-19) caused by severe acute respiratory syndrome coronavirus 2 (SARS-CoV-2) appeared unexpectedly and abruptly in December 2019 in Wuhan. Shortly, this infectious disorder represented a global health problem of great impact on the lifestyle of various populations (e.g., during and after the lockdown period) [2] and on their ability to heal themselves. The easy transmissibility of SARS-CoV-2 quickly resulted in the suspension or sharp reduction of various ophthalmic activities considered non-urgent, including cataract surgery or some intraocular injections [3,4].

## 2. An Ongoing Paradigm Shift for Ocular Surgery during the COVID-19 Era

Despite the imperative need to continuously practice eye surgery to avoid the abovementioned problems, there are currently little-shared and vague recommendations among the various countries on safety in operating rooms for health care workers (HCWs) and patients, as well as poor legal protection for surgeons (potentially transmitting the coronavirus).

Clearly, different considerations must be made according to the type of surgery, i.e., elective or urgent.

The American Academy of Ophthalmology (AAO), i.e., the world’s largest association of eye physicians and surgeons, recommended on 18 March 2020 that all ophthalmologists stop immediately any treatment other than urgent or emergent care, thus including both office-based care and surgical care [5]. This issue was based on recommendations from the American College of Surgeons and the Centers for Disease Control and Prevention (CDC). However, the final decision on what to define as “urgency” was left to the doctor’s judgment, who always had to take into account the social and medical circumstances of the individual patients.

Thereafter, some facilities have begun to prepare for the resumption of elective surgeries based on the 16 April guidelines from the federal government [6]. Thus, the academy considered the possibility of reopening ophthalmology care based on the flattening of the curve, and recommended a path of local opening based on the situation in each area (and no longer national). Again, AAO announced the future publication of documents detailing how to reopen more normal practices in the COVID-19 era [7], inviting each ophthalmologist to make individual decisions about protocols for the use of personal protective equipment (PPE) and other measures. Anyhow, the academy recommended protection for the mouth, nose (e.g., an N95 mask), and eyes (e.g., goggles or shield) and slit-lamp breath shields when caring for patients potentially infected with SARS-CoV-2 [8]. For eye clinics, social distancing, frequent disinfection of rooms, and the wearing of face masks by both patients and caregivers were mandatory. All these roles implied a reduction in the volumes of patients to be visited and treated surgically compared to the pre-pandemic period, with longer turnover times between cases.

In other parts of the world with different socioeconomic needs and contexts (e.g., India, China, or Africa), the reference scientific societies and scholars advised similar protective behaviors [9,10,11].

Of note, the British Oculoplastic Surgery Society (BOSS) highlighted a whole series of ocular surgical procedures associated with the generation of bioaerosol (i.e., with high biological risk of COVID-19 transmission), in particular the lacrimal procedures. Of course, other surgeries involving the nasal or sinus cavities, open suctioning, and orbital surgery using power/electrical instruments should also require special attention [12].

Curiously, the Royal College of Ophthalmology recommended a reduction of the length of the surgical procedures by indicating only the most experienced and older surgeons as potential operators (who are at increased risk for serious age-related complications from COVID-19), thus excluding younger surgeons from the operating room (with possible repercussions on their training courses) [13]. Clearly, this interim guideline may lead to an ethical and scientific paradox over time.

## 3. Controversial Issues for Eye Surgeons, Patients, and Public Health

Overall, the suggested PPE for eye surgery may obviously cause a number of changes and technical difficulties for surgeons. As a result, there may be greater complexity in focusing due to goggles (with the impossibility of using a face shield), a larger and more uncomfortable distance between the operator and microscope, a different sensitivity to the foot pedal due to overshoes, and discomfort and/or skin irritation due to facial masks. Clearly, double disposable sterile surgical gloves, suggested for some surgical procedures, can also change the sensitivity of the surgeon [12].

With this respect, the use of three-dimensional (3-D) heads-up display systems for both anterior segment and vitreoretinal surgery [14,15] may help to reduce part of these drawbacks. In fact, the surgeon can perform microsurgical procedures not by looking at the eyepieces of the microscope but by viewing the microscopic image on a panel display sent from a 3-D camera. In this way, the distance between the surgeon and patient increases, and goggles or a face shield may be less mandatory.

Clearly, the preventive measures taken for the various types of surgical procedure imply different scenarios. As regards the impact of surgery on eye health, in case of a prolonged duration of the pandemic, the continuous postponement of elective surgery inevitably facilitates a progressive deterioration of visual function in general population.

On the other hand, in urgent or emergency surgical procedures, since the onset of the pandemic, eye surgeons have quickly encountered the dilemma of how to protect patients and themselves from potential infections (indeed, many procedures produce bioaerosol and therefore can spread the virus in the environment of operating rooms), and how to legally shield themselves against potential sue. These situations of risk of contagion, and the doubtful indication of when to operate or not to operate, have created many difficulties during the COVID-19 era, e.g., the complex risk/benefit assessment of each surgical procedure. In addition, there is currently also an enigma for surgeons to be faced with an asymptomatic/pre-symptomatic or with a false negative (COVID-19 tests) patient.

From a theoretical point of view, it would be desirable to identify effective protocols in preventing COVID-19, with specific guidelines for each surgical specialty. In Ophthalmology, for instance, pre-surgical conjunctival and nasopharyngeal tests (swabs) may help in screening cases requiring special attention in addition to pre-surgical and post-surgical drug prophylaxis. Although there is no confirmed effective agent for post-exposure prophylaxis, as stated by the National Institutes of Health “Coronavirus 2019 (COVID-19) Treatment Guidelines” (updated 4/21/2020) [16], it may be useful (in the meantime to have robust scientific evidence) to execute irrigation with 5% povidone iodine also at the end of eye surgery, and in the following days, eventually in conjunction with an ophthalmic preparation containing 0.2% benzalkonium chloride. This treatment may also be valuable for surgeons and nurses, whose ocular surfaces become accidentally exposed to air droplets or secretions from patients [17].

In any case, a responsible utilization of all personal protective equipment available for surgeons and patients, and sanitization of rooms are the best prophylactic tools available today.

## 4. Ethic Concerns and Medical Liability

Among the various problems related to the COVID-19 pandemic, there are ocular surgeons’ concerns regarding the potential medical liability of the care due to scarce scientific evidences on the adequate sanitization of operating room during procedures, or related to general protection for patients.

Similar concerns have been previously discussed by policy makers regarding the limited availability of means to appropriately treat patients in different contexts [18].

Thus, some institutional organizations in the world have provided different levels of medical liability protection, though the latter was rather limited. 

Accordingly in the USA, for example, Congress under the Coronavirus Aid, Relief, and Economic Security Act (“CARES Act”) offered legal protection to volunteer HCWs during the ongoing pandemic [19], while some States extended liability protection to employed or contracted HCWs through state orders [20]. In the first case, the exemption from liability under state or federal law included any damage caused by an act or omission, except for some obvious circumstances (e.g., negligence, misconduct, conscious indifference, criminal conduct, alcohol or intoxicating drugs use etc …). However, non-volunteers HCWs were not included in this provision. In the second case, several States, e.g., New York, have provided immunity from civil liability to some HCWs for any damage resulting from an act or omission, except for gross negligence.

On the other hand, no specific protective provisions have been granted to HCWs in many other Countries around the world to protect them from potential medical liability.

For example, in Italy, although there is a law proposal aimed at limiting penal liability, ordinary law is currently in force yet. To note, despite healthcare facilities are the places where infectious occur most frequently, the current law on the crime of “negligent epidemic” (art. 438, penal code) does not provide for exceptions or mitigations for HCWs, potentially implying different scenarios for an act or omission [21], and the Prosecutors at the head of some of the more important judicial districts promoted investigations in this direction, looking for medical misconducts. 

Clearly, medical needs and scientific knowledge are changing rapidly with regards to the SARS-CoV-2 epidemic, the related treatments and tests, as well as the responses of policy makers, thus leading to ongoing variations in interim recommendations from scientific societies. Although it is not possible to entirely eliminate the risk of infection (thus obtaining the desirable zero rate) during the ongoing pandemic, prudent adjustments to the indicated precautions may be warranted [22,23].

Overall, guidelines adapted in this pandemic era may help protect clinical ophthalmologists and eye surgeons should anyone question the therapy decisions made during the ongoing pandemic. But as always in a medical context, guidelines represent only a flexible tool to address a clinical issue on the best available evidences, needing always a personalized implementation on the basis of personal, structural, and organizational skills and knowledge.

## 5. Conclusions

While telemedicine has been able to solve some problems in clinical ophthalmology, the lack of adequate and precise scientific/legal indications for surgeons and patients could result in an excessive reduction in the volume of surgical interventions during a pandemic era and in the immediately following period. Additionally, the weakness and slowness of the health system may lead to the inadmissible inability to meet real needs and ensure health care to all patients.

Now, the need to clarify as soon as possible the abovementioned points is therefore imperative. Otherwise, COVID-19 could remain a dilemma for eye surgeons until the discovery of effective and safe protocols for prevention and therapy, reliable point-of-care testing, and validated methods for sanitizing the operating room.

## References

[1] World Health Organization (2014). Visual Impairment and Blindness.

[2] Napoli P.E., Nioi M. (2020). Global Spread of Coronavirus Disease 2019 and Malaria: An Epidemiological Paradox in the Early Stage of a Pandemic. J. Clin. Med..

[3] Napoli P.E., Nioi M., d’Aloja E., Fossarello M. (2020). The Ocular Surface and the Coronavirus Disease 2019: Does a Dual ‘Ocular Route’ Exist?. J. Clin. Med..

[4] Napoli P.E., Nioi M., Mangoni L., Gentile P., Braghiroli M., d’Aloja E., Fossarello M. (2020). Fourier-Domain OCT Imaging of the Ocular Surface and Tear Film Dynamics: A Review of the State of the Art and an Integrative Model of the Tear Behavior During the Inter-Blink Period and Visual Fixation. J. Clin. Med..

[5] American Academy of Ophthalmology (2020). Recommendations for Urgent and Nonurgent Patient Care.

[6] U.S. Federal Government and the Center for Disease Control (2020). Guidelines for Opening Up America Again DC. White House Plan for Opening up America Again. https://www.whitehouse.gov/openingamerica/.

[7] American Academy of Ophthalmology (2020). Returning to Ophthalmology Practice.

[8] American Academy of Ophthalmology (2020). Important Coronavirus Updates for Ophthalmologists.

[9] All India Ophthalmological Society Coronavirus and Eye Safety.

[10] Yu A.Y., Tu R., Shao X., Pan A., Zhou K., Huang J. (2020). A comprehensive Chinese experience against SARS-CoV-2 in ophthalmology. Eye Vis..

[11] The International Agency for the Prevention of Blindness Online Conference on Ophthalmology and COVID-19 in African Units. https://www.iapb.org/news/online-conference-on-ophthalmology-and-covid-19-in-african-units/.

[12] British Oculoplastic Surgery Society (2020). Update Guidance on Infection Risk and Personal Protective Equipment (PPE) for Oculoplastic Surgery during the COVID-19 Pandemic.

[13] The Royal College of Ophthalmology (2020). COVID-19 Clinical Guidance for Ophthalmologists.

[14] Moura-Coelho N., Henriques J., Nascimento J., Medeiros M.D. (2019). Three-dimensional Display Systems in Ophthalmic Surgery–A Review. Eur. Ophthalmic Rev..

[15] Eckardt C., Paulo E.B. (2016). Heads-up surgery for vitreoretinal procedures: An experimental and clinical study. Retina.

[16] U.S. National Library of Medicine (2020). Post-Exposure Prophylaxis/Preemptive Therapy for SARS-Coronavirus-2 (COVID-19 PEP).

[17] Kampf G., Todt D., Pfaender S., Steinmann E. (2020). Persistence of coronaviruses on inanimate surfaces and their inactivation with biocidal agents. J. Hosp. Infect..

[18] Cohen I.G., Crespo A.M., White D.B. (2020). Potential legal liability for withdrawing or withholding ventilators during COVID-19: Assessing the risks and identifying needed reforms. JAMA.

[19] Section 3215, Coronavirus Aid, Relief and Economic Security Act, Pub. L. 116–136. https://www.congress.gov/116/bills/hr748/BILLS-116hr748eas.pdf.

[20] Health Care and Life Sciences Client Alert COVID-19: New York Governor Temporarily Suspends Various Health Care Rules and Requires Health Care Facilities to Increase Bed Capacities. https://www.ebglaw.com/news/covid-19-new-york-governor-temporarily-suspends-various-health-care-rules-and-requires-health-care-facilities-to-increase-bed-capacities/.

[21] Codice Penale Italiano, Libro II, Titolo VI, Capo II, Art. 438. https://www.brocardi.it/codice-penale/libro-secondo/titolo-vi/capo-ii/art438.html.

[22] Adams J.G., Walls R.M. (2020). Supporting the health care workforce during the COVID-19 global epidemic. JAMA.

[23] Feldman O., Meir M., Shavit D., Idelman R., Shavit I. (2020). Exposure to a Surrogate Measure of Contamination From Simulated Patients by Emergency Department Personnel Wearing Personal Protective Equipment. JAMA.

